# BioLuminescent OptoGenetics in the choroid plexus: integrated opto- and chemogenetic control *in vivo*

**DOI:** 10.1117/1.NPh.11.2.024210

**Published:** 2024-06-28

**Authors:** Eric Klein, Sophie Marsh, Jordan Becker, Mark Andermann, Maria Lehtinen, Christopher I. Moore

**Affiliations:** a Brown University, Providence, Rhode Island, United States; b Beth Israel Deaconess Medical Center Harvard, Boston, Massachusetts, United States; c Boston Children’s Hospital, Boston, Massachusetts, United States

**Keywords:** BioLuminescent, optogenetic, chemogenetic, dynamics, behavior

## Abstract

**Significance:**

The choroid plexus (ChP) epithelial network displays diverse dynamics, including propagating calcium waves and individuated fluctuations in single cells. These rapid events underscore the possibility that ChP dynamics may reflect behaviorally relevant and clinically important changes in information processing and signaling. Optogenetic and chemogenetic tools provide spatiotemporally precise and sustained approaches for testing such dynamics *in vivo*. Here, we describe the feasibility of a novel combined opto- and chemogenetic tool, BioLuminescent-OptoGenetics (BL-OG), for the ChP *in vivo*. In the “LuMinOpsin” (LMO) BL-OG strategy, a luciferase is tethered to an adjacent optogenetic element. This molecule allows chemogenetic activation when the opsin is driven by light produced through luciferase binding a small molecule (luciferin) or by conventional optogenetic light sources and BL-OG report of activation through light production.

**Aim:**

To test the viability of BL-OG/LMO for ChP control.

**Approach:**

Using transgenic and Cre-directed targeting to the ChP, we expressed LMO3 (a *Gaussia* luciferase-VChR1 fusion), a highly effective construct in neural systems. In mice expressing LMO3 in ChP, we directly imaged BL light production following multiple routes of coelenterazine (CTZ: luciferin) administration using an implanted cannula system. We also used home-cage videography with Deep LabCut analysis to test for any impact of repeated CTZ administration on basic health and behavioral indices.

**Results:**

Multiple routes of CTZ administration drove BL photon production, including intracerebroventricular, intravenous, and intraperitoneal injection. Intravenous administration resulted in fast “flash” kinetics that diminished in seconds to minutes, and intraperitoneal administration resulted in slow rising activity that sustained hours. Mice showed no consistent impact of 1 week of intraperitoneal CTZ administration on weight, drinking, motor behavior, or sleep/wake cycles.

**Conclusions:**

BL-OG/LMO provides unique advantages for testing the role of ChP dynamics in biological processes.

The choroid plexus (ChP) is a vascular-epithelial network that extends throughout the ventricular system. The ChP is heterogeneous in its genetic composition, with substantial variation between ventricular sub-compartments and across the lifespan.^1^^,^^2^ We recently observed that the ChP exhibits rapid calcium dynamics in awake mice *in vivo*, including spreading waves and cell-specific fluctuations.^3^ These rapid and longitudinal dynamics, and their regional specialization, suggest that the ChP plays distinct roles in processing signals to impact downstream targets and potentially behavior. The known functions of the ChP include processing cerebrospinal fluid (CSF) and forming the blood-CSF barrier, transmitting (or blocking) signals moving between the body and brain. Performance of these functions, and others not yet delineated, may be dynamic, reflecting ChP-centered information-processing as observed in other body networks.

Studying neural network dynamics has benefitted from a recent surge in new techniques for controlling genetically selected cell types with spatio-temporal precision or sustained modulation. These methods include chemically activated modulation of biophysical state (chemogenetics) and light-driven stimulation of focally expressed opsins (optogenetics). The most common chemogenetic approach, DREADDs, modulates cell excitability for hours through G-protein coupled receptors.^4^^,^^5^ While effective in several applications, the relatively long timescale of a single DREADDs modulation, and potential off-target effects created by its activator, are both considerations in experimental design.^6^^–^^8^ Optogenetic approaches permit cell-specific and millisecond precision, allowing direct testing of phenomena such as neural synchronization in behavior^9^^,^^10^ but are less effective for sustained control and typically require invasive implants to provide optical access. Another key limitation of both approaches is that they are mediated by different constructs. In many experimental settings, a combined ability to probe the same preparation with both sustained activation of all cells expressing a given genotype, and focal control of distinctly target foci, would be highly advantageous.

Here, we describe the key enabling steps for use of BioLuminescent-OptoGenetics (BL-OG), a multi-functional control approach, in testing the potentially dynamic role of epithelial ChP. In the BL-OG strategy, a luciferase enzyme and opsin fusion molecules, and therefore in the same cell (Intraluminescence^11^^–^^13^) or in different cells (interluminescence; e.g., across an “optical synapse”^14^). When a small molecule luciferin is presented, chemically generated photon production drives the opsin: a chemogenetic strategy. The same opsin is also available to conventional optogenetic drive by external light.

The use of bioluminescence, as compared to other forms of light administration to drive opsins, has multiple advantages.^15^ First, bioluminescence has evolved within multiple types of living organisms, with expression in the majority of marine species.^16^^,^^17^ As such, its “components” are typically highly bio-compatible and have already been used extensively in biomedical research.^18^^–^^20^ An additional benefit to the BL-OG strategy is that light production indicates the timing and relative intensity of luciferin activation of its target,^21^ and photon emission therefore provides a direct report of the timing and relative intensity of chemogenetic engagement. To optimize proximity between photon production to its target, luciferases and opsins are often tethered by a short amino acid chain, a “LuMinOpsin” construct (LMO).^12^ While multiple LMO variants now exist to meet distinct experimental needs,^11^^,^^22^^–^^26^ the most widely used instantiation to date is LMO3, a relatively bright *Gaussia* luciferase (sbGluc) tethered to Volvox ChannelRhodopsin (VChR1).^27^ The LMO3 construct has proven effective for chemogenetially driving cortical, spinal, midbrain, and peripheral neurons in developing and mature mice.^27^^–^^33^ As such, they are potentially ideal for many key research directions in ChP study requiring long timescale modulation, including potential roles in development and long-term plasticity.

As a key first step in enabling the control and study of ChP dynamics with these tools, we expressed LMO3 in epithelial ChP using a transgenic approach. We found robust bioluminescent output *in vivo* with LMO3 activation in the ChP with introduction of the luciferin Coelenterazine (CTZ). This activation was observed following several different routes of administration, with rapid, bright output following intravenous (IV) and intracerebroventricular (ICV) application, and long-time constant activation with intraperitoneal (IP) delivery. We next tested whether chronic repeated CTZ presentation, key for longitudinal studies, caused any gross behavioral effects. In LMO3 in ChP or littermate controls, we did not detect any changes in weight, basic indices of motor activity or sleep-wake cycling across seven days of administration. Together, these initial studies indicate BL-OG can provide a robust, multi-functional strategy for studying subtle effects of ChP dynamics *in vivo*.

## 1 Results

### 1.1 Distinct Routes of BL-OG Activation Provide Distinct Activation Profiles for ChP Study

**Fig. 1:**
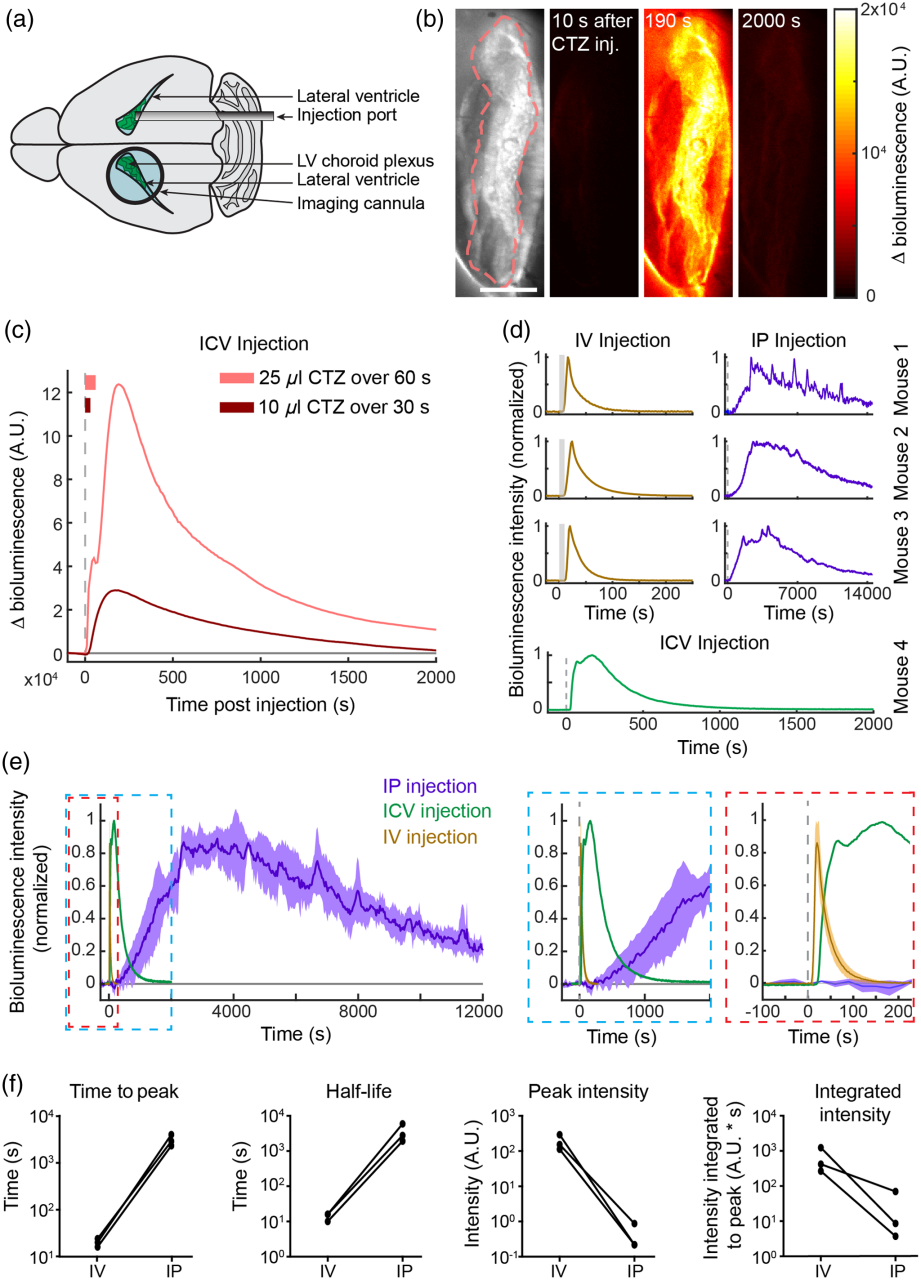
BL-OG in ChP enables multiple time courses of chemogenetic control. (a) Diagram of visualization area. (b) Example of BioLuminescent photon output with ICV luciferin delivery. (c) Example of distinct photon output in the same mouse with two different luciferin injections. (d) Individual time courses for IV (fast), IP (sustained), and ICV BioLuminescent activation. (e) Mean BioLuminescent activation in all conditions. (F) Comparison of IV and IP activation metrics in 3 mice.

While LMO3 has been effectively activated *in vivo* in a variety of neural cell types and at different developmental time points, this construct has not been tested in ChP epithelium, and differences resulting from the local milieu (e.g., pH) or cell-type specific expression, could render its bioluminescent recruitment inactive. To test its functionality, and determine the time course and relative amplitude of LMO3 bioluminescent activation depending on the method of *in vivo* CTZ injection, we compared three different routes of delivery: intraperitoneal (IP), intravenous (IV), and intracerebroventricular (ICV) injection. Coelenterazine (CTZ) was administered to FoxJ1-Cre:LSL-LMO3-eYFP mice, which show strong expression in ChP epithelial cells.^3^^,^^29^^,^^34^ In all testing, mice were implanted with an imaging cannula^3^ that allows optical access to the left lateral ventricle and ChP. We recorded ChP bioluminescent output through the canula in anesthetized mice (isofluorane .5-1%) using an Andor iXon Ultra 888 EMCCD with a 5X, 0.14 NA objective (Mitutoyo). Because the imaging cannula precluded direct vertical placement of the ICV delivery cannula into the same lateral ventricle, we targeted the injection cannula to the contralateral lateral ventricle by an oblique trajectory through the dorsal posterior cortical surface [diagrammed in Fig. 1(a) and described in the Appendix (Sec. 3)].

The three modes of administration, ICV, IV, and IP, each provided a distinct pattern and timing of response onset. With ICV administration, bioluminescent photon output began 10 to 30 s following injection onset and progressed from center outward, in a pattern spatially consistent with a route of CTZ diffusion from the contralateral ventricle, through the third ventricle, to the ChP in the imaging field. Activated tissue continued to peak bioluminescence over tens of seconds and then faded slowly to the minimal observed effect [Fig. 1(b)]. To test the impact of rate and volume of CTZ injections on ChP bioluminescence, in one mouse we injected CTZ, 25  μl over 60 s, and 10  μl over 30 s, into the lateral ventricle. A fourfold difference in magnitude between peak bioluminescent response was observed between the two conditions [Fig. 1(c), light versus dark red curves]. A bioluminescent response of similar amplitude and time constant to that following the 10  μl to 30 s injection was produced in a second animal from a trial with a 5  μL to 20 s ICV injection [Fig. 1(d), bottom green trace].

Prior studies have shown that IV luciferin injections drive a faster rise time and higher peak photon production than IP injections.^35^^–^^37^ We next tested whether these principles hold for BL-OG in the unique circulatory and chemical environment of the ventricle.^38^ We imaged anesthetized LMO3 in ChP mice following IV or IP CTZ injection (N=3 mice). Figure 1(d) (left) shows responses normalized as percent of maximal emission for trials from each animal in IP or IV injection conditions and a plot from an additional animal in an ICV trial for comparison (bottom). Initial bioluminescent output onsets ∼10  s following the start of IV injection [Fig. 1(d), gold]. In contrast, initial activity after IP injection was significantly slower in onset and rise time, and varied widely in ascent, on the scale of hundreds of seconds, as observed in prior studies (purple).^37^ Also, unlike ICV or IV injections, where response curves comprised a rapid initial climb to peak and then a smooth exponential decay, bioluminescent output curves resulting from IP injections demonstrated local sub-peaks and troughs in photon production in all mice [see Figs. 1(d) and 1(e), purple].

Figure 1(e) shows the difference in temporal progression of the response between IP, ICV, and IV trials, plotted as the average percent of maximal response across subjects for each condition. The blue and red dashed expansions show the relative time course onset for the three injection types, showing IV onset within seconds of injection, an intermediate rise time for ICV injection, and the slow onset and rise time with IP injection. While the IP response begins later, it persists much longer than the other administration routes, on the scale of tens of thousands of seconds: we did not observe a return to baseline within any IP imaging session. To quantify these temporal response differences, we calculated the time-to-peak for each animal across trial types. In all cases, responses to IV injection peaked <25  s after onset, whereas IP injection responses peaked from 2000 to >4000  s after onset [Fig. 1(f), left]. Considering the post-peak half-maximal response, all IV trials decayed to half-maximal <20  s after maximal effect, whereas IP trials decayed to half-maximal from 2000 to 6000 s.

To compare response magnitude, we normalized data across trial-types for imaging exposure (0.5 Hz IV, 0.33 Hz IP frame rate), and across individuals for small differences in mg/Kg of CTZ introduced by our standard injection in an individual. We found ∼100-fold higher peak emission on IV versus IP trials. To describe the differences in total light emission across trial types, we integrated normalized bioluminescence responses from injection to peak-effect timepoints. Although IP responses to CTZ injection peaked and persisted considerably longer than IV responses, integrated responses from IV injections showed one to two orders of magnitude greater total output [Fig. 1(F)].

### 1.2 Repeated CTZ Administration Does Not Impact Basic Health Indices

**Fig. 2:**
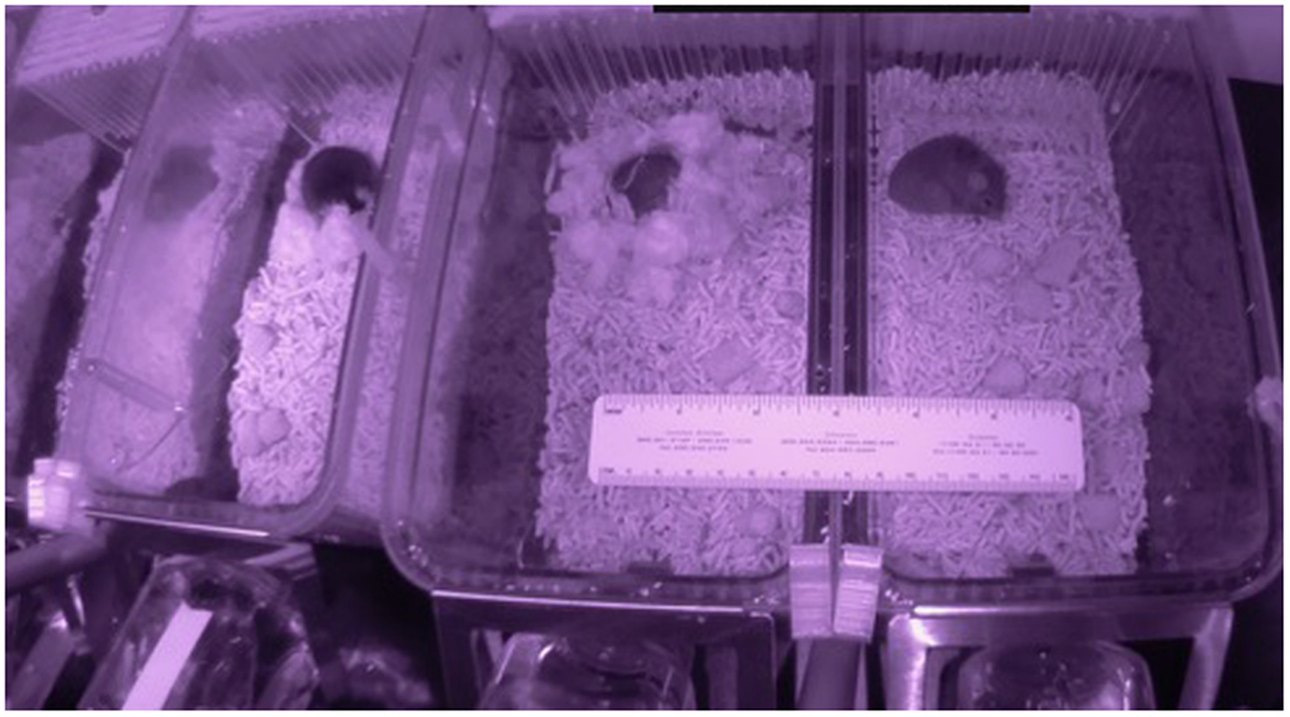
Example frame from video obtained for multi-mouse automated assessment of the impact of 1 week of CTZ injections. Please see text for further details.

As a further enabling step in the use of BL-OG for ChP research, we tested whether repeated CTZ administration or LMO3 expression in the ChP impacts basic indices of health or behavior. To obtain systematic video for analysis, we designed and laser cut *de novo* plexiglass cage tops for Thoren split cages, using novel three-dimensional (3D) printed clamps. Video cameras were held on adjacent tripods, one split cage/camera. Data were taken with Raspberry Pi 4B 2G MakerFocus Night Vision Cameras with Adjustable Focus (5MP HD Wide Angle Fisheye Lens OV5647 1080p; two cameras had a mechanically shuttered IR filter). Mice were kept on a reverse light:dark cycle. Head, body, and tail identification points were trained in DeepLabCut to analyze mouse location, movement, and orientation and then placed into real space through a custom-written Cartesian transformation.^39^ Figure 2 shows a sample image obtained in the dark cycle (ruler: 6 in./15 cm).

Mice (N=12) were monitored for seven days following daily intraperitoneal (IP) injection of CTZ (75  μl on d1-4, 100  μl on d5-6, and 110  μl on d7 at 2.36 mM in saline; prolume coelenterazine *in vivo*) or vehicle control. Double-transgenic mice were generated by crossing the FoxJ1-Cre and LSL-LMO3 (“LMO3 in ChP” mice: N=6; Jax Laboratories Strain #: 034853; see Ref. 21 for details). The remaining N=6 were C57/Bl6 mice without LMO3 expression (non-LMO3). For each group, half (N=3) were assigned to receive either CTZ or vehicle (LMO3 in ChP given CTZ, one female; control CTZ, 1F; LMO3 in ChP vehicle, 2F; control vehicle, 2F). Each mouse was weighed and checked for signs of discomfort or lethargy daily, video monitored in their home cages and housed under a 12/12 LD cycle to record estimates of wakefulness, movement, and drinking behaviors.

**Fig. 3:**
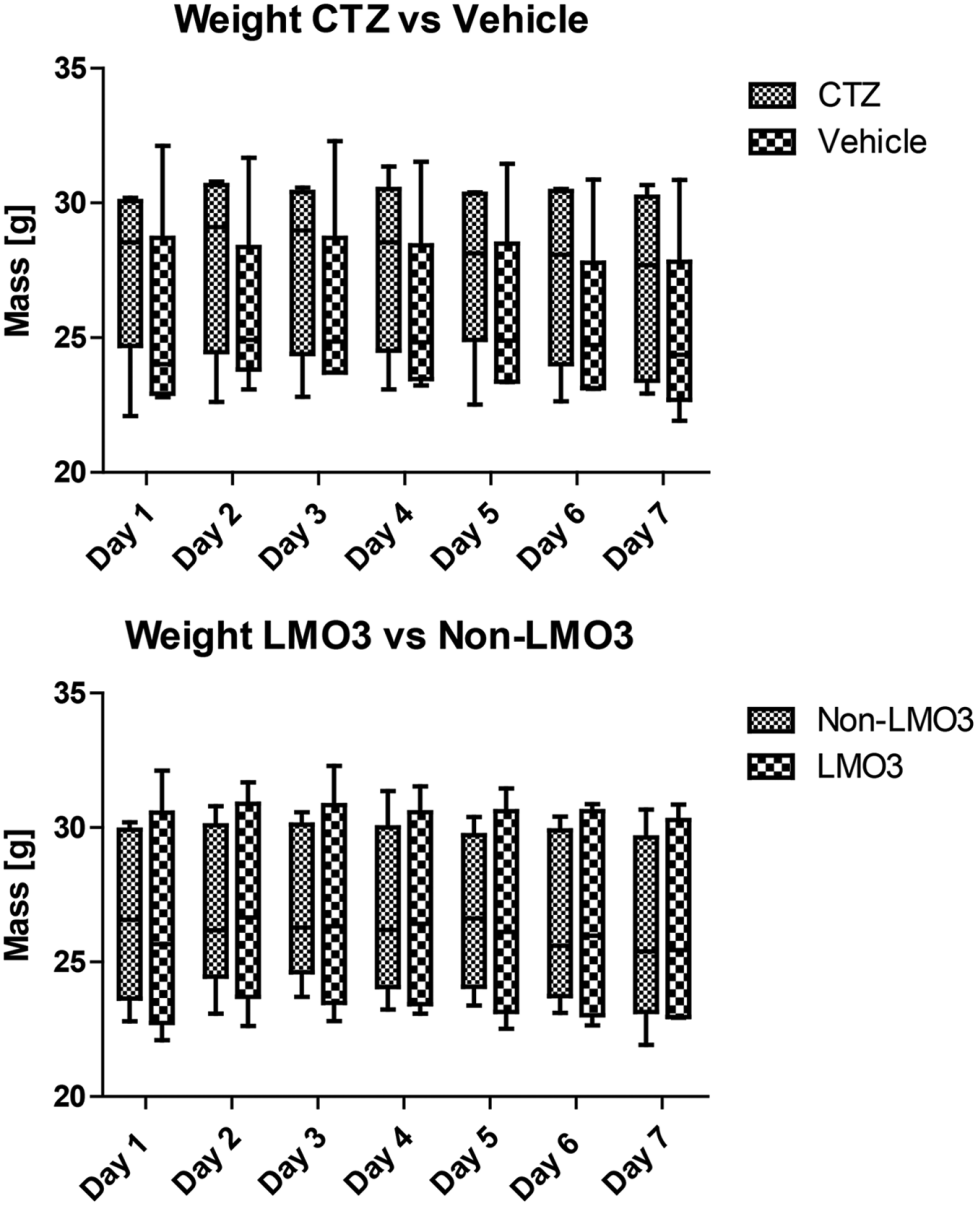
Repeated CTZ administration and transgenic expression of LMO3 in ChP did not impact weight across days. Across several metrics, mice did not show any adverse effect of CTZ administration or expression of LMO3 in the ChP. Please see accompanying text for details.

Across d1-7 both mice in the CTZ and vehicle group showed robust health. No mice showed signs of discomfort (e.g., porphyrin secretions), diminished grooming, or failed startle responses. Across days, mice in the CTZ and vehicle groups maintained stable body weights (Friedman rank sum tests; CTZ $\chi^{2}=10.21$, p>0.05; vehicle $\chi^{2}=13.16$, p<0.05, all Bonferroni corrected pairwise comparisons p>0.05; Fig. 3). No significant differences in total weight change, day 1 to day 7, were found between luciferin groups (CTZ versus vehicle) nor between genotype groups (ChP-LMO3 versus non-LMO3; Wilcoxon Mann-Whitney test, p>0.05; Fig. 3).

Mice in all groups displayed normal phasic home cage behaviors, as assessed by observation and quantified by videography obtained on days 4 to 7. During the light (resting) phase of the circadian cycle, mice in all groups initiated more sleep bouts per monitored hour, and slept for more total time, than in the dark (waking) phase (light M=9.35, SD=0.85, dark M=5.60, SD=0.58, Wilcoxon rank sum test n=12, p<0.05). Mice also initiated more movement bouts in the dark cycle (light M=526.05, SD=53.85, dark M=754.30, SD=48.31, Wilcoxon rank sum test n=12, p<0.05), moved at greater average velocity per bout (light M=1.21, SD=0.06, dark M=1.54, SD=0.23  cm per second, Wilcoxon rank sum test n=12, p<0.05). All these behaviors were stable and did not drift significantly day-to-day.

No effects were observed between CTZ and vehicle in sleep or movement metrics in the lights on or in the lights off epochs (Wilcoxon rank sum test, p>0.05 for all comparisons). For LMO3 in ChP versus non-ChP mice, no difference was found in sleeping bouts in the lights off epoch (Wilcoxon rank sum test, p>0.05). We did observe a modest, if significant, difference in sleep bouts in the lights on condition, with LMO3 in ChP showing a greater number (p=0.026; LMO3 in ChP 9.85±0.83 versus non-ChP 8.86±0.53, mean ± standard deviation). However, this finding was not observed in the other metrics of sleep persistence, as there were no difference observed in sleep time or movement bouts between these groups in these epochs (Wilcoxon rank sum test, p>0.05 for all comparisons).

## 2 Discussion

There is an increasing appreciation of the role of non-neural networks in creating adaptive behavior through their dynamics: In this regard, the ChP is, for example, implicated in memory formation^40^ and in setting behavioral state through the distribution of molecular signals.^41^ Dynamics governing the ongoing activity of the ChP is also implicated in a variety of diseases, including Alzheimer’s^42^ and hydrocephalus.^43^ In all these domains, hypothesis testing is served by the ability to control detailed ChP biophysical state in targeted ways, and with varying time courses and degrees of temporal precision.

Here, we show the viability of a novel approach, BL-OG, that provides both chemogenetic and optogenetic control options.^12^^,^^21^ Expression of this construct constitutively in ChP did not detectably alter weight or behavior. Further, administration of the enabling chemogenetic agent, the luciferin CTZ, also did not detectably perturb these metrics. Further, we found that a variety of routes of CTZ administration are viable for driving light generation in LMO3 in ChP mice, with distinct intensities and time constants best suited to distinct experimental conditions. In sum, this strategy is well-positioned for use to investigate the ChP.

In this study, we did not have sufficient power to reach any significant conclusions regarding the recruitment of LMO3 in the ChP on health or behavior. Beyond the statement that gross changes such as marked hyperactivity or mortality were not evident, it remains for future studies to apply the tools enabled here to test the impacts of subtle, temporary changes in epithelial ChP dynamics on health and/or behavior.

## 3 Appendix: Methods

All animal care and experimental procedures were approved by the Marine Biological Laboratory and the Brown University Institutional Animal Care and Use Committees. Animals were singly housed on a 12-h light/dark cycle with standard mouse chow and water provided *ad libitum*. Experiments were performed on adult mice of both sexes (6f) and aged between 13 and 52 weeks. In the imaging studies, N=5 mice were employed. In the behavioral assays, N=12 mice were employed.

Mice were prepared for bioluminescence imaging in a manner similar to that used for imaging of calcium activity and immune cells in the ventricle in Ref. 3 (see also Ref. 44). Briefly, FoxJ1-Cre:LSL-LMO3-eYFP mice (n=5) were anesthetized with 2% Isoflurane in oxygen, delivered at 1.5  L/min, and body temperature was maintained at 37°C. The scalp was shaved, and an incision was made to remove skin covering the dorsal skull including the posterior frontal bone, parietal bone, and interparietal bone. A steel head-bar was fixed to the skull using Metabond dental adhesive. A 3-mm diameter craniotomy was performed, centered at −0.8  mm AP, −2.0  mm ML relative to Bregma. The underlying cortex was aspirated by suction using a 23 gauge blunt canula to a depth of ∼2.0  mm, allowing access to the top of the lateral ventricle. A 3.0-mm diameter cannula with a glass coverslip glued at the tip was inserted into the craniotomy and fixed in place with Metabond.

For experiments involving ICV delivery of CTZ, we inserted a second cannula into the lateral ventricle contralateral to the imaging cannula. Skulls were initially marked for drilling along the slanted track of cannula insertion using a custom 3D-printed template positioned over the posterior interparietal bone. A burr hole was then drilled at the correct entry point. The template was used to guide the tip of a 9.5 mm, 24 gauge cannula though the burr hole in the posterior to anterior direction with a 8.39 deg downward angle from horizontal to reach the lateral ventricle target location of −0.58  mm AP, 1.2 mm ML, and 2.0 mm DV relative to Bregma. The cannula was fixed to the posterior skull with Metabond^®^ dental adhesive and capped. Animals were given a time-release NSAID (Meloxocam SR) for post-surgical analgesia and allowed to recover in their home cages for greater than 14 days before use in experiments.

### 3.1 *In Vivo* Bioluminescent Imaging

Mice were initially anesthetized with 2% isoflurane in oxygen delivered at 1.5  l/min and then moved into a light-tight enclosure for imaging. Animals were secured in the imaging environment by clamping their head-bar to steel posts (Thor Labs) and isoflurane concentration was reduced to 0.5% to 1% to maintain anesthesia for the course of the experiment. An Andor iXon Ultra EM-CCD camera with a 5×, 0.14 NA objective (Mitutoyo) was focused on the ChP through the animal’s imaging cannula and window. Images were viewed and recorded with Andor Solis software. For all experiments, we performed injections of water-soluble CTZ (Prolume) diluted in deionized water (2.36 mM). Injections of CTZ were delivered by a Quintessential Stereotaxic Injector syringe pump (Stoeling). For ICV injections, CTZ was loaded into a syringe attached to catheter tubing terminating in a 26-gauge cannula (WPI). ICV caps were removed from the head-restrained, anesthetized mouse, and the 26-gauge cannula was gently threaded into the ICV cannula. For all IP and IV injections, CTZ-loaded syringes were attached to catheters terminating in a 29 gauge needle (Instech). The needle was used to cannulate either the lateral tail-vein (IV) or peritoneum (IP) and was then held in place with surgical tape.

### 3.2 Imaging Bioluminescence During ICV Injection of CTZ

Imaging data were recorded from two mice with ICV implants for CTZ administration. Baseline images were acquired for a duration of 2 min. Images were recorded at a rate of 0.1 or 0.5 Hz in the first and second mouse, respectively. After baseline recording, controlled injection of CTZ was initiated. Post-injection, images were recorded for an additional 33 min. Following recording, anesthesia was discontinued, mice regained consciousness and were returned to their home cage. See text above for other relevant information.

### 3.3 Imaging Bioluminescence During IV and IP Injection of CTZ

Imaging data were recorded from three mice, each of which underwent IP and IV injections of CTZ on separate days. Images were acquired at a rate of 0.5 Hz for IV trials and 0.33 Hz for IP trials, respectively. Baseline images were acquired for 2 min (IV trials) or 3 min (IV trials). Following baseline recording, controlled injection of CTZ was initiated. 75  μL of CTZ was injected over a 10 s duration in all IV trials. For IP trials, injections of 310, 350, or 400  μL were delivered over a 10 s duration. Following injection, images were recorded for an additional 4.03 h. Following recording, anesthesia was discontinued, mice regained consciousness, and were returned to their home cage.

### 3.4 Analyses of Bioluminescence Imaging Data

Images were analyzed using Matlab (Mathworks). For all trials, regions of interest (ROIs) were created to assess overall bioluminescence emanating from the ChP by tracing a mean projection image [Fig. 1(b), left panel]. Pixel values recorded before exposure to CTZ (zero bioluminescence): Those in the ChP were selected as in the top 93% of intensity to form a mask baseline ROI. Difference images were created by subtracting the ROI image at each time point from this baseline ROI image. Pixels from each difference image were averaged to create a timeseries of changes in bioluminescence from baseline for each trial, in arbitrary units.

## Data Availability

A video showing BioLuminescent Choroid Plexus activation can be found in the Brown Digital Repository (https://doi.org/10.7301/Z0BK19VB). Behavioral data are available upon request.
